# *Thymus vulgaris* alleviates UVB irradiation induced skin damage *via* inhibition of MAPK/AP‐1 and activation of Nrf2‐ARE antioxidant system

**DOI:** 10.1111/jcmm.12968

**Published:** 2016-09-19

**Authors:** Zhengwang Sun, Sang Yong Park, Eunson Hwang, Mengyang Zhang, Seul A. Seo, Pei Lin, Tae‐Hoo Yi

**Affiliations:** ^1^ Department of Oriental Medicinal BiotechnologyCollege of Life ScienceKyung Hee University Global CampusYongin‐siGyeonggi‐doKorea

**Keywords:** *Thymus vulgaris*, UVB, MAPK, AP‐1, Nrf2

## Abstract

Solar ultraviolet (UV) radiation‐induced reactive oxidative species is mainly responsible for the development of photoageing. Rosmarinic acid was one of the main bioactive components detected in *Thymus vulgaris* (TV) we extracted. In this study, UVB‐induced skin damages have been shown to be ameliorated by treatment with TV in hairless mice (HR‐1) skin, demonstrated by decreased matrix metalloproteinases (MMPs) and increased collagen production. However, the underlying molecular mechanism on which TV acted was unclear. We examined the photoprotective effects of TV against UVB and elucidated the molecular mechanism in normal human dermal fibroblasts. *Thymus vulgaris* remarkably prevented the UVB‐induced reactive oxygen species and lactate dehydrogenase. Dose‐dependent increase in glutathione, NAD(P)H: quinone oxidoreductase1 and heme oxygenase‐1, by TV was confirmed by increased nuclear accumulation of Nrf2. Furthermore, 5‐Methoxyindole‐2‐carboxylic acid was introduced as a specific inhibitor of dihydrolipoyl dehydrogenase (DLD). We demonstrated that Nrf2 expression was regulated by DLD, which was a tricarboxylic acid cycle‐associated protein that decreased after UVB exposure. Besides, TV significantly diminished UVB induced phosphorylation of mitogen activated protein kinases pathway, containing extracellular signal‐regulated kinase, Jun N‐terminal kinase and p38, which consequently reduced phosphorylated c‐fos and c‐jun. Our results suggest that TV is a potential botanical agent for use against UV radiation‐induced oxidative stress mediated skin damages.

## Introduction

Solar ultraviolet (UV) radiation consist of UVA (320–400 nm), UVB (280–320 nm) and UVC (100–280 nm), which induces cutaneous lesions such as photoageing and photo‐carcinogenesis. Particularly UVB is mainly responsible for the development of oxidative stress, inflammation and DNA damage resulting in photoageing, characterized by wrinkling, dryness, sagging and thickening 1, 2. Ultraviolet irradiation induces reactive oxygen species (ROS), such as singlet oxygen, superoxide anion, hydroxyl radical and hydrogen peroxide, which in turn regulate varieties of cellular functions including matrix metalloproteinases (MMPs) secretion and collagen destruction 3. The booming MMPs production and subnormal extracellular matrix (ECM) level were observed in premature skin ageing 4. Matrix metalloproteinases play a pivotal role in degrading collagens and elastin in the ECM, which are involved in the dermal strength and resiliency 5. Therefore, antioxidants possessing ROS scavenging ability are promising photoprotective agents.

Researches have been reported that accumulation of ROS could regulated the activation of antioxidant genes and transcription factors to counteract oxidative stress 6. One of the major employed defense system is the nuclear factor E2‐releated factor 2 (Nrf2)‐antioxidant‐response element (ARE) signalling pathway 7. Under quiescence, the Kelch‐like ECH‐associated protein 1 (Keap‐1) sequesters Nrf2 and subjected to proteasome for degradation in the cytoplasm. Under stimulation, Nfr2 translocates into nucleus, where it binds to the ARE, initiating the expression of cytoprotective enzymes and antioxidants, such as NAD(P)H: quinone oxidoreductase1 (NQO‐1), heme oxygenase‐1 (HO‐1), and glutathione 8. Dihydrolipoyl dehydrogenase (DLD) is the only tricarboxylic acid (TCA) cycle‐associated protein that decreased by UVB irradiation, and the reduced DLD level may be associated with oxidative stress in the mitochondria. Namely, the main function of UV‐targeted DLD in the skin may be antioxidant, meaning that DLD could protect skin damage caused by UV‐induced oxidative stress 9. In this article, a specific reversible inhibitor of DLD, 5‐Methoxyindole‐2‐carboxylic acid (MICA) was used to investigate the role of DLD played in the Nrf2‐ARE antioxidant system.

Furthermore, UV‐induced oxidative stress is proved to regulate the phosphorylation of protein kinases *via* varieties of cascades, such as mitogen activated protein kinases (MAPKs) and activated protein (AP‐1) signalling pathway 10, 11. Antioxidants were reported to attenuate the activation of MAPK signalling 12, 13, 14, and this journal also signifying that the MAPKs are potential objects of ROS for treatment of UV‐induced skin damage. The green tea polyphenols prevented UV induced oxidative damage and MMPs expression in keratinocytes and mouse skin by its antioxidant and immunomodulatory effects 15, 16. Dietary administration of proanthocyanidins extracted from grape seed suppressed UVB induced skin pigmentation and photo‐carcinogenesis and in animal model 17, 18.

*Thymus vulgaris* (TV) also called thyme is a traditional used herb rich in rosmarinic acid. The anti‐inflammatory, analgesic and antipyretic activities of *Thymus serphyllum* L. were evaluated in mice 19. The methanol extracts of thyme showed antioxidant and antimicrobial activities *in vitro* 20. However, there is no report of TV on UVB irradiation induced photodamage. In this study, we demonstrated that TV protected the UVB‐induced photodamage in hairless mice (SKH: HR‐1). We elucidated the protective mechanisms against UVB induced damage *via* DLD‐Nrf2‐ARE, MAPK and AP‐1 signalling in normal human dermal fibroblasts (NHDFs).

## Materials and methods

### Chemicals

ELISA kits for MMP‐1, IL‐6, and TGF‐β1 were purchased from R&D Systems (R&D Systems, Inc., Minneapolis, MN, USA), while ELISA kits for procollagen type I were purchased from Takara (Takara, Shiga, Japan). DMEM, FBS and penicillin streptomycin were purchased from Gibco BRL (Grand Island, NY, USA). MICA was used a specific inhibitor of DLD. Dry TV leaves were purchased from mountain rose herbs company (Eugene, OR, Florida, USA). Unless otherwise mentioned, solvents were purchased from Samchun Chemicals (Seoul, Korea), and inorganic salts were from Sigma‐Aldrich (St. Louis, MO, USA).

### Sample preparation

The dried TV (10 g) were powdered and extracted three times with 1000 ml of ethyl alcohol (50%) for 24 hrs at room temperature. The ethyl alcohol was filtered and subjected to vacuum evaporation at 38°C.

### High performance liquid chromatography analysis

High performance liquid chromatography (HPLC) was performed on a Dionex Chromelon TM chromatography data system with P580 and UVD100 detectors (Thermo Fisher Scientific Inc., Waltham, MA, USA). Chromatographic separation was performed on a Waters Sunfire C_18_ column (250 × 4.6 mm, 5‐μm particle size). Elution was performed with a methanol/acetonitrile (3:1) gradient containing 1% formic acid. The gradient was linearly increased from 10% to 90% methanol over 35 min. The injection volume was 10 μl and the flow rate was 1 ml/min.

### Animal UV irradiation and sample treatment

The animal experimental protocol for this study [KHUASP(SU)‐12‐09] was approved by the Institutional Animal Care and Use Committee of Kyung Hee University. Seven‐week‐old male albino hairless mice (HR‐1) (20–27 g) were obtained from Central Lab Animals, Inc. (Seoul, Korea). UVB irradiation was performed as described previously 10. Hairless mice were randomly divided into five groups of five mice per cage: Normal (without UVB irradiation); Control (UVB irradiation); Positive control (UVB irradiation + retinyl palmitate (RP)); TV 1% (UVB irradiation + 1% TV); TV 5% (UVB irradiation + 5% TV). In the topical application group, mice were exposed to 100 mJ/cm^2^ UVB radiation seven times per week for the first week, and 100 mJ/cm^2^ three times a week for 4 weeks thereafter. However, the normal group was not exposed to UVB radiation.

### Wrinkle measurement

Light‐bodied silicone (SilfloR, Flexico, Colchester, UK) was introduced to obtain replicas of the dorsal skin. The visiometer technique was introduced to detect changes in the transparency of thin silicone replicas. Photos were obtained with a CCD video camera and analysed using Skin Viscometer SV 600 software (Courage & Khazaka, Cologne, Germany). The R1, R2, R3, R4 and R5 values were obtained as previously described 10.

### Histological analysis

Biopsies (dorsal skin) were obtained, fixed in 4% paraformaldehyde, dehydrated in ethanol and then embedded in paraffin. Approximately 10‐μm‐thick sections were deparaffinized and stained with haematoxylin and eosin and Masson's trichrome staining. Stained slides were then photographed using a light microscope (ZEISS Observer D2; Zeiss, Munich, Germany).

### Cell culture, UVB irradiation and sample treatment

Normal human dermal fibroblasts (MCTT Core, Inc., Seoul, Korea) were cultured with DMEM (supplemented with 10% heat‐inactivated FBS and 1% penicillin‐streptomycin). The cells were maintained at 37°C in a humidified atmosphere containing 5% CO_2_. Then, sub‐confluent cells were subjected to irradiation supplied by a Bio‐Link BLX‐312 (Vilber Lourmat GmbH, Marne‐la‐Vallee France) with 144 mJ/cm^2^ for NHDFs 10. Cells were treated with TV (1, 10 and 100 μg/ml) and control cells were kept quiescent. After 72 hrs, supernatants were harvested to assess the production of MMP‐1, MMP‐3, TGF‐β1 and IL‐6. In the case of RT‐PCR, cells were harvested 24 hrs after UVB irradiation. No more than 10 cell passages were used in the experiments.

### MTT assay

Cell viability tests were performed as described previously 10. In brief, MTT(3‐(4,5‐Dimethylthiazol‐2‐l)‐2,5‐Diphenyltetrazolium Bromide) (100 μg/ml) was added and incubated for another 2 hrs after 72 hrs of sample treatment, then DMSO was added in order to dissolve the formazan crystals. The absorbance was read at 570 nm using a microplate reader (Molecular Devices E09090; San Francisco, CA, USA).

### Measurement of DPPH radical and ROS scavenging ability

2,2‐diphenyl‐1‐picrylhydrazyl (DPPH) was introduced to measure the antioxidant ability of TV extract. Ascorbic acid was used as a positive control.

Following UVB (144 mJ/cm^2^) irradiation and samples treatment for 24 hrs, NHDFs were stained with 30 μM 2′7′‐dichlorofluorescein diacetate (DCFH‐DA; Sigma‐Aldrich) for 30 min. at 37°C. The cells were then rinsed twice with PBS and subjected to a multi‐mode microplate reader (Molecular Devices Filter Max F5).

### Monitoring of lactate dehydrogenase release

The lactate dehydrogenase (LDH) level in the culture medium was measured using an LDH cytotoxicity assay kit (Roche Diagnostics, Mannheim, Germany). This method is based on the LDH‐catalysed reduction in pyruvate to lactate by NADH. Briefly, equal amounts of culture supernatant were mixed with fresh LDH buffer containing NADH. After a 30‐min. incubation at room temperature, the absorbance was measured with an ELISA microplate reader at 490 nm.

### Determination of intercellular GSH

Total GSH content in the culture medium was determined using a GSH assay kit (Cayman Chemical Co, Ann Arbor, MI, USA). The kit uses an enzymatic recycling method involving glutathione reductase to quantify GSH. The sulphydryl group of GSH reacts with DTNB (5,5′‐dithiobis‐2‐nitrobenzoic acid, Ellman's reagent) and produced a yellow‐coloured compound, 5‐thio‐2‐nitrobenzoic acid (TNB). The absorbance was measured with at 405 nm. The rate of TNB production indicates the concentration of GSH in the sample based on the standard curve provided with the assay kit.

### Detection of DLD

After exposure to UVB, NHDFs were cultured with MICA (1 mM) or TV (10 and 100 μg/ml) for 24 hrs. Total cell lysates were assayed by Western blot according to our previous research 10.

### Preparation of cytosolic and nuclear extracts

After exposure to UVB, NHDFs were treated with TV (10 and 100 μg/ml) for 3 hrs. The cytosolic and nuclear portions of the cells were separated with a commercial kit (NE‐PER nuclear and cytoplasmic extraction reagents; Pierce).

### Measurement of MMP‐1, MMP‐3, cytokines IL‐6, procollagen type I and TGF‐β1

We measured the concentrations of MMP‐1, MMP‐3, IL‐6, procollagen type I and TGF‐β1 in the medium with commercially available ELISA kits according to the manufacturer's instructions. Each sample was analysed in triplicate.

### Reverse transcription‐PCR

At the indicated time, RNA from UVB‐irradiated NHDF cells was isolated using Trizol reagent according to the manufacturer's instructions (Invitrogen Life Technologies, Carlsbad, CA, USA). Reverse transcription (RT)‐PCR were performed in a Veriti Thermal Cycler (Applied Biosystems, Foster City, CA, USA) according to our previous research 10. PCR products were stained with ethidium bromide and separated by 2.0% agarose gel. Each experiment was repeated at least three times.

### Western blot analysis

Total tissue and cell lysates were assayed by Western blot according to our previous research 10. The antibodies Nrf2, Histione, extracellular signal‐regulated kinase (ERK), phosphor‐ERK, Jun N‐terminal kinase (JNK), phosphor‐JNK, p38, phosphor‐p38, anti‐rabbit‐HRP, anti‐goat‐HRP and anti‐mouse‐HRP were from Cell Signaling Technology (Danvers, MA, USA), and β‐actin, c‐fos, phosphor‐c‐fos, c‐jun, phosphor‐c‐jun, MMP‐1, procollagen type I, TGF‐β1, elastin and DLD‐E3 were purchased from Santa Cruz Biotechnology (Dallas, TX, USA). Each experiment was repeated at least three times.

### Statistical analysis

The data are presented as means ± S.D values of three independent experiments in triplicate. Statistical analysis was performed using one‐way anova test. Statistical significance was set at *P* < 0.05.

## Results

### Analysis of extracts from TV

**Figure 1:**
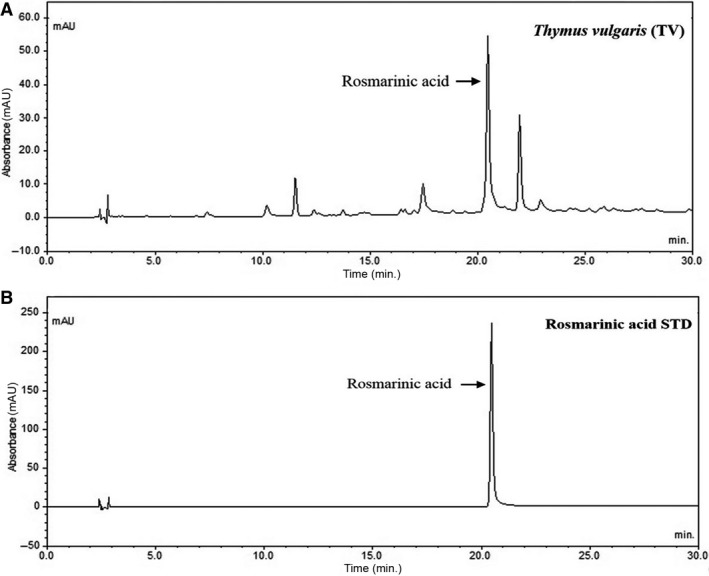
HPLC results. (**A**) TV seed extract; (**B**) Standard of rosmarinic acid.

To prepare TV samples, we extracted the dried seeds of TV (10 g) with 50% ethyl alcohol, obtaining crude product (1.46 g) with a 14.6% yield. As shown in Figure 1, the main compound rosmarinic acid was detected, which comprised about 27.3% of the TV extract (Fig. 1).

### Inhibition effects of TV on wrinkle formation and collagen digestion in UVB exposed hairless mice skin

**Figure 2:**
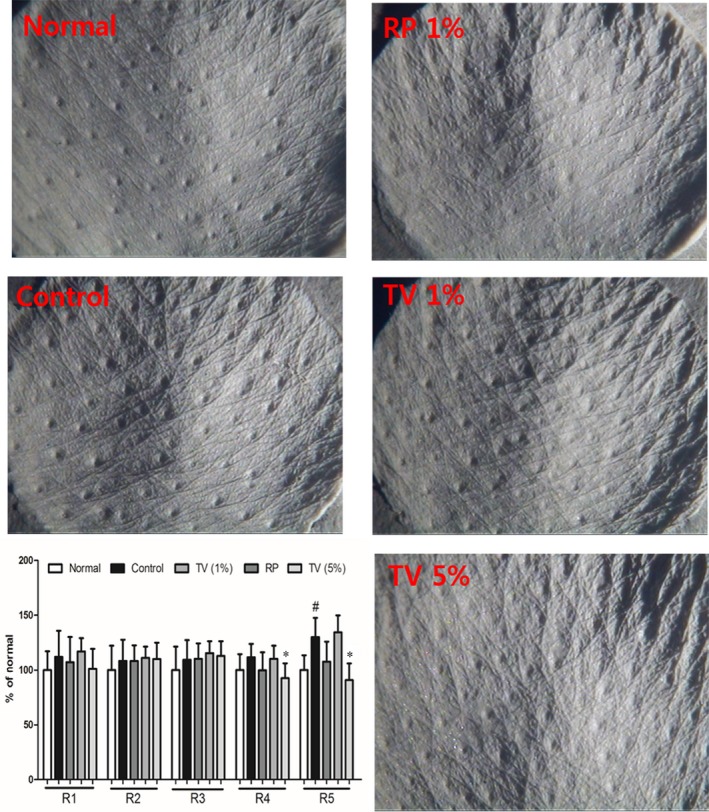
Wrinkle measurement and analysis of skin replicas. Photographs of replicas: Normal (without UVB irradiation); Control (UVB irradiation); RP 1% (UVB irradiation + 1% RP); TV 1% (UVB irradiation + 1% TV); TV 5% (UVB irradiation + 5% TV). Arbitrary units (R1 – R5) were assigned to each sample based on depth measurements of the furrows according to shadow size and brightness: skin toughness (R1), largest value of the five distances (R2), average of maximum distance (R1) derived from each of the five parts of the line (R3), mean area surrounded by horizontal line drawn at the highest crest and furrows profile (R4), and mean deviation of the furrows profile to the middle line (R5). All data are shown as the mean ± S.D. of at least three independent experiments performed in triplicate. #*P* < 0.05, compared to the control; *P < 0.05, compared to the group receiving only UVB radiation.

Skin Wrinkle formation was measured in hairless mice chronically exposed to UV‐B radiation for 8 weeks. Integumentary images and cutaneous characteristics for wrinkle were taken and analysed by digital and CCD optical camera. Long‐term UVB irradiation accelerated wrinkle formation in the mouse skin, rendering the skin rough and scaly, indicative of skin photoageing. When TV (1% and 5%) was topically applied to the dorsal skin of hairless mice, the wrinkle formation was noticeably attenuated and the dorsal skin attributes were improved (Fig. 2).

**Figure 3:**
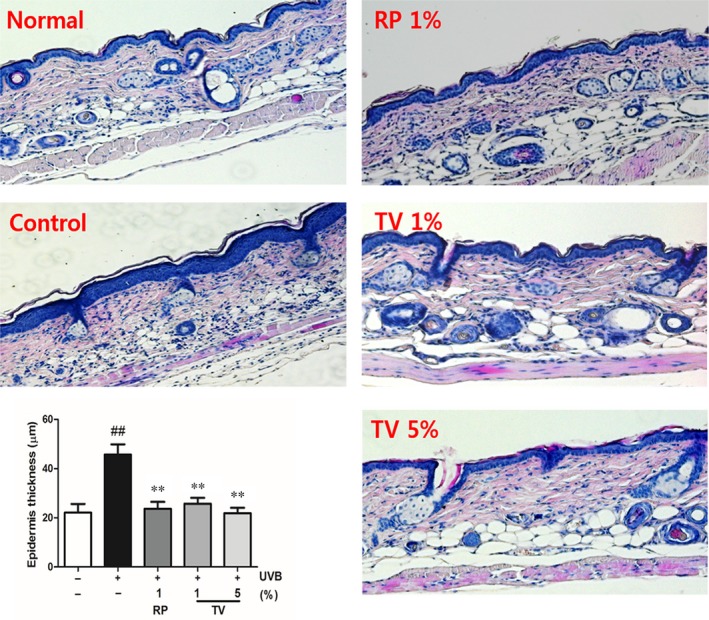
Haematoxylin and eosin‐stained sections (100 times magnification) photomicrographs of topically treated hairless mice dorsal skin. Normal (without UVB irradiation); Control (UVB irradiation); RP 1% (UVB irradiation + 1% RP); TV 1% (UVB irradiation + 1% TV); TV 5% (UVB irradiation + 5% TV). All data are shown as the mean ± S.D. of at least three independent experiments performed in triplicate. ##*P* < 0.01, compared to the control; **P < 0.01, compared to the group receiving only UVB radiation.

Haematoxylin and eosin stained slides were photographed (ZEISS Observer D2, Germany) and skin epidermal thickness was measured (Axio Vision Rel.4.8) (Fig. 3). As shown, the epidermal thickness of the dorsal skin increased 106.7% due chronic UVB exposure, compared with that of normal group. Topical application of TV (1% and 5%) substantially decreased the epidermal thickness of the dorsal skin, which were 44.5% and 53.3%, respectively, compared with that of UVB irradiated control group. Therefore, topical treatment of TV diminished roughness and wrinkles formation caused by chronic UVB irradiation.

**Figure 4:**
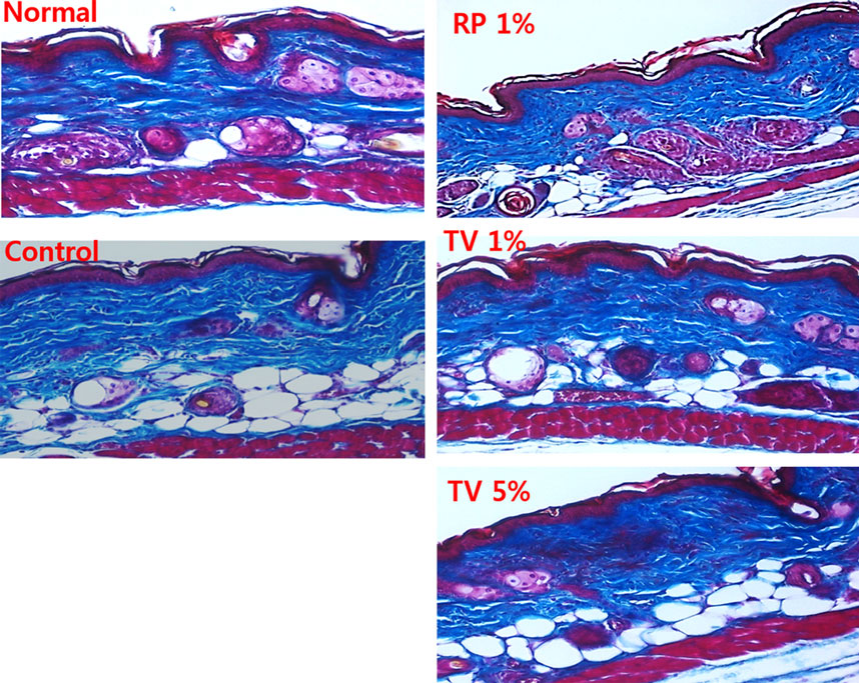
Masson's trichrome‐stained sections (200 times magnification) photomicrographs of topically treated hairless mice dorsal skin. Normal (without UVB irradiation); Control (UVB irradiation); RP 1% (UVB irradiation + 1% RP); TV 1% (UVB irradiation + 1% TV); TV 5% (UVB irradiation + 5% TV).

Histological staining with masson‐trichrome for dermal collagen fibres showed that chronic UVB irradiation damaged the cellular collagen density in the dermis. In contrast, there was a heavy staining of collagen fibres in TV (1% and 5%) treated hairless mice exposed to UVB, compared with that of UVB irradiated control group (Fig. 4).

### Effects of TV on the production of MMP‐1, elastin, procollagen type I and TGF‐β1 in UVB exposed hairless mice skin

**Figure 5:**
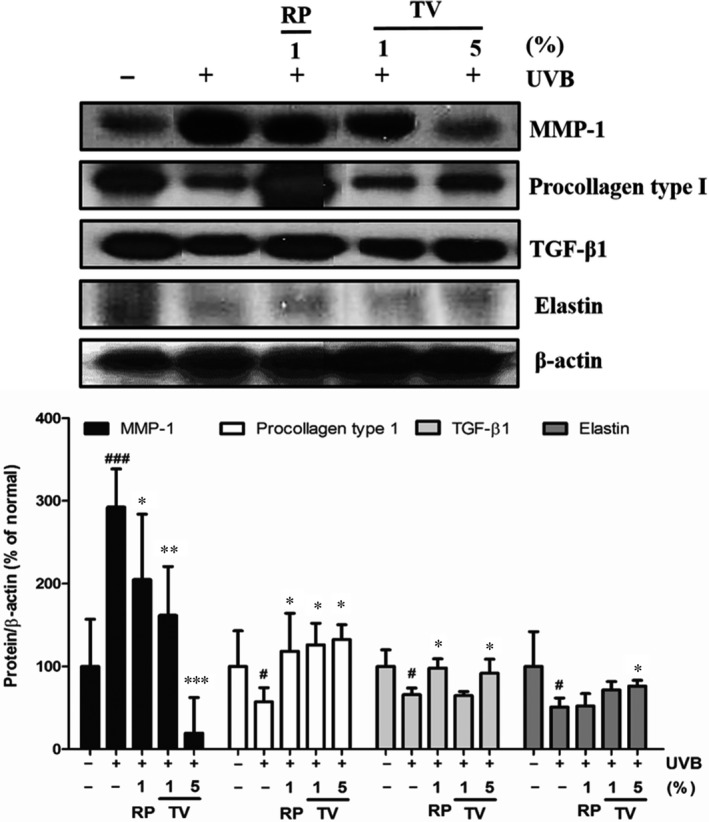
Protein expressions of MMP‐1, procollagen type I, TGF‐β1 and elastin in dorsal skin of hairless mice. Signal intensities were quantified and normalized to the corresponding value of β‐actin. Values are reported as mean ± S.D. #*P* < 0.05 *versus* normal control, *P < 0.05 *versus* UVB‐irradiated control. All data are shown as the mean ± S.D. of at least three independent experiments performed in triplicate. #*P* < 0.05, ###*P* < 0.001, compared to the control; *P < 0.05, **P < 0.01, ***P < 0.001, compared to the group receiving only UVB radiation.

As the structural protein of ECM, procollagen type I and elastin were degraded by MMP‐1 while stimulated by TGF‐β1. In our study, UVB irradiation caused 184.6% increase in MMP‐1 in the UVB irradiated group, compared with that of normal group. When TV (1% and 5%) was topically applied, the MMP‐1 level was alleviated in a dose‐dependent manner, which were 42.8.1% and 89.3%, respectively, compared with that of UVB irradiated control group (Fig. 5).

Conversely, Decreased procollagen type I caused by UVB was reinstated by 133.3% and 116.7% in the topically administrated with 1% and 5% TV groups, respectively. Similarly, UVB reduced elastin by 49.2%, however, with topical application of TV (1% and 5%), the impaired elastin was increased by 41.2% and 49.1%, respectively, compared with that of UVB irradiated group. In the case of TGF‐β1, the decreased protein was also significantly restored by topical application of TV (5%), which was increased by 39.4% compared with that of UVB irradiated control group (Fig. 5).

### Toxicity analysis of TV extract on NHDFs

**Figure 6:**
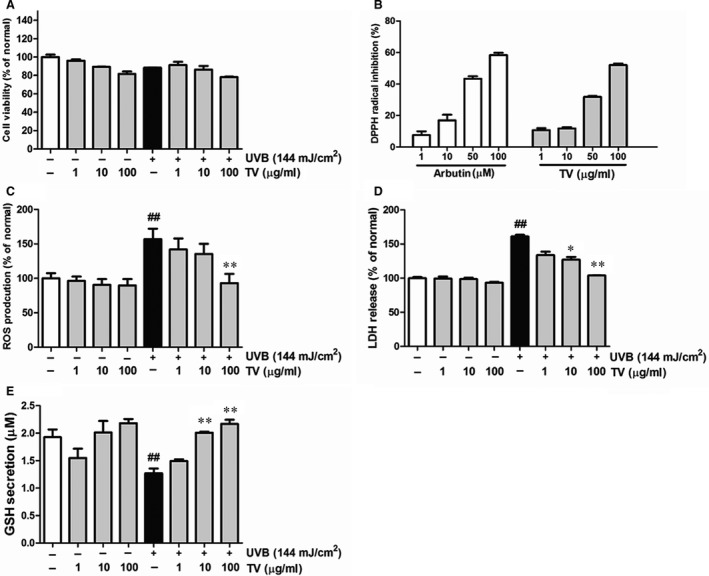
Protective effects of TV on NHDFs. NHDFs were irradiated or non‐irradiated with UVB, followed by treatment with the indicated concentrations of TV (1, 10 and 100 μg/ml) for 72 hrs. (**A**) Cell viability; (**B**) ROS production; (**C**) LDH release; (**D**) GSH secretion. All data are shown as the mean ± S.D. of at least three independent experiments performed in triplicate. ##*P* < 0.01, compared to the control; *P < 0.05, **P < 0.01, compared to the group receiving only UVB radiation.

MTT analysis was performed to measure viabilities of NHDFs. After subjecting cells to UVB, the cell viability was reduced by 13.7%. When cells were treated with the indicated concentrations (1, 10 and 100 μg/ml) of TV extract, cell viability was reduced at higher concentrations (10 and 100 μg/ml), but there was not significantly difference compared with non‐treated group (Fig. 6A).

### Cytoprotective effects of TV on UVB‐irradiated NHDFs

DPPH radical scavenging ability was measured to determine the antioxidant ability of TV. As shown in Figure 6B, ascorbic acid was introduced as a positive control, and TV extract (100 μg/ml) scavenged 57.2% of DPPH radical (Fig. 6B).

It is well known that UV induces ROS, causing oxidative stress, which is harmful to cells. NHDFs exposure to UVB showed significant increase in ROS generation, whereas this trend was reduced by 40.8% when treated with TV (100 μg/ml) in UVB‐induced cells (Fig. 6C). Besides, exposure of NHDFs to UVB markedly elevated the release of LDH, whereas TV (1, 10, 100 μg/ml) treatment reduced LDH release in a dose‐dependent manner, almost close to the control values at 100 μg/ml (Fig. 6D). Moreover, the intracellular GSH was remarkably depleted because of UVB irradiation, whereas TV treatment restored GSH in a dose‐dependent manner. Specifically, TV (100 μg/ml) restored GSH level by 70.4%, compared with the UVB irradiation control (Fig. 6E).

### Promotion of DLD and Nrf2 nuclear translocation and antioxidants in UVB‐irradiated NHDFs

**Figure 7:**
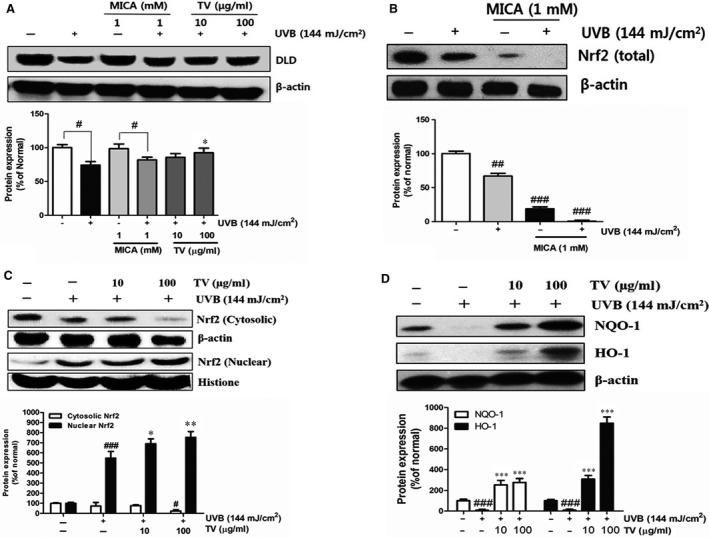
Effects of TV on DLD, Nrf2, HO‐1 and NQO‐1 expression in UVB‐irradiated NHDFs. (**A**) NHDFs were irradiated or non‐irradiated with UVB, followed by treatment with MICA (1 mM) or TV (10 and 100 μg/ml) for 24 hrs, the expression of DLD was detected by western blot; (**B**) NHDFs were irradiated or non‐irradiated with 144 mJ/cm^2^, followed by treatment with MICA (1 mM) for 24 hrs, the expression of total Nrf2 was detected by western blot; (**C**) NHDFs were irradiated or non‐irradiated with 144 mJ/cm^2^, followed by treatment with TV (10 and 100 μg/ml) for 2 hrs, both the cytosolic and nuclear Nrf2 were detected by western blot; (**D**) NHDFs were irradiated or non‐irradiated with 144 mJ/cm^2^, followed by treatment TV (10 and 100 μg/ml) for 2 hrs, the expression of HO‐1 and NQO‐1 was detected by western blot. β‐actin and histone were used as an internal control, respectively. The band intensities were quantified, normalized and calculated as the percentage of the basal response. The results are shown as the mean ± S.D. of at least three independent experiments performed in triplicate. #*P* < 0.05, ###*P* < 0.001, compared to the control; *P < 0.05, **P < 0.01, ***P < 0.001, compared to the group receiving only UVB radiation.

To investigate the role of DLD played in ameliorating UVB‐induced oxidative stress, MICA was introduced as a specific inhibitor of DLD. UVB irradiation impaired the DLD expression, while TV (100 μg/ml) enhanced its production by 24.6%, compared with the UVB irradiation control (Fig. 7A). With the treatment of MICA, the total production of Nrf2 was quenched (Fig. 7B).

To assess the antioxidant mechanism of TV acted, both cytosolic and nuclear Nrf2 proteins and antioxidants were assessed in UVB‐irradiated NHDFs. UVB irradiation induced the production of Nrf2 in nucleus. Simultaneously, nuclear amount of Nrf2 was significantly increased by 30.2% with the treatment of TV (100 μg/ml), compared with the UVB irradiation (Fig. 7C). Similarly, HO‐1 and NQO‐1 level was dramatically elevated with the treatment of TV (Fig. 7D).

### Blocking of MMPs and cytokine IL‐6 production and degradation procollagen type I and TGF‐β1 in UVB irradiated NHDFs

**Figure 8:**
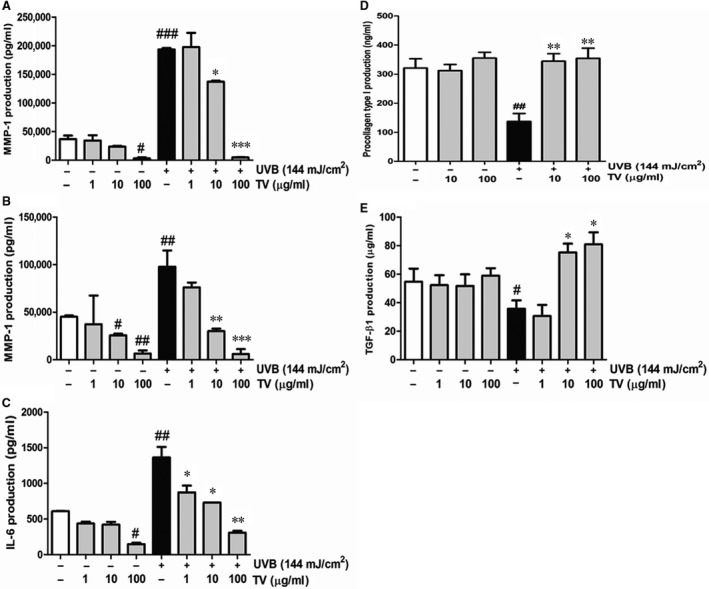
Effects of TV on MMP‐1, MMP‐3, procollagen type I, IL‐6 and TGF‐β1 secretion in UVB irradiated NHDFs. NHDFs were irradiated or non‐irradiated with UVB, followed by treatment with the indicated concentrations of TV (1, 10 and 100 μg/ml) for 72 hrs. (**A**) MMP‐1 production; (**B**) MMP‐3 production; (**C**) IL‐6 production (**D**) Procollagen type I production; (**E**) TGF‐β1 production. All data are shown as the mean ± S.D. of at least three independent experiments performed in triplicate. #*P* < 0.05, ##*P* < 0.01, ###*P* < 0.001, compared to the control; *P < 0.05, **P < 0.01, ***P < 0.001, compared to the group receiving only UVB radiation.

Matrix metalloproteinases is a major collagenolytic enzyme responsible for collagen damage in UV‐irradiated human skin 10. Compared with the normal group, both MMP‐1 and MMP‐3 levels in NHDFs were markedly elevated because of UVB irradiation. When added TV extract, the trend was reversed. Specifically, TV quenched MMP‐1 secretion by 31.6% at 10 μg/ml and 73.7% at 100 μg/ml, and suppressed MMP‐3 production by 74.5% at 10 μg/ml and 93.6% at 100 μg/ml in NHDFs (Fig. 8A and B). Similarly, the pro‐inflammatory cytokine IL‐6 was severely irritated as a result of the UVB irradiation. By contrast, treatment with TV significantly calmed the activation of IL‐6 proteins. The inhibition rates were 46.3% at 10 μg/ml, and 75.7% at 100 μg/ml respectively (Fig. 8C).

On the other hand, this study attempted to confirm whether TV restored the collagen and TGF‐β1 breakdown of NHDFs caused by UVB. As expected, TV dose‐dependently enhanced the secretion of procollagen type I and TGF‐β1, which were dampened by UVB irradiation. Specifically, TV promoted the production of procollagen type I by 151.7% at 10 μg/ml and 158.4% at 100 μg/ml. In the case of TGF‐β1, those was 114.3% at 10 μg/ml and 128.6% at 100 μg/ml in NHDFs (Fig. 8D and E).

### Effects of TV on the mRNA expression of MMP‐1 and procollagen type I in UVB irradiated NHDFs

**Figure 9:**
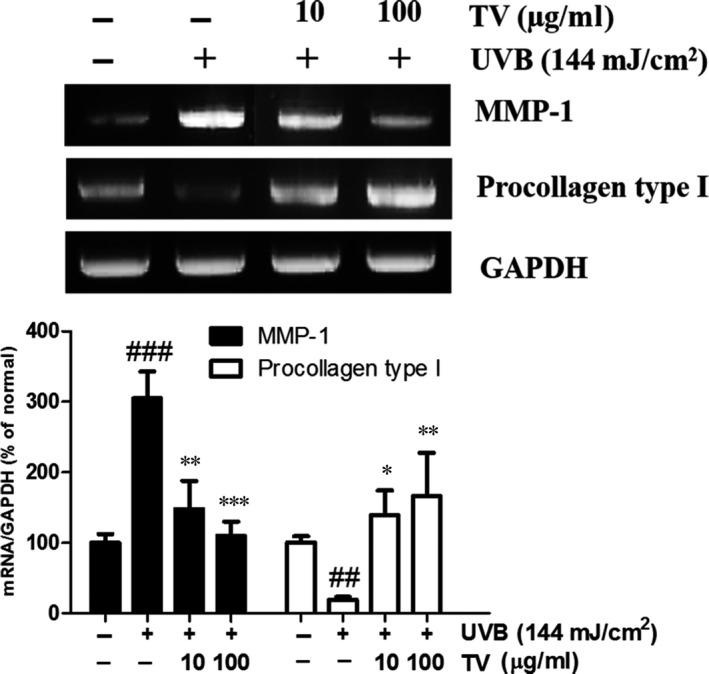
Effects of TV on the mRNA expression of MMP‐1 and procollagen type I in UVB irradiated NHDFs. NHDFs were irradiated with UVB followed by treatment with TV (10 and 100 μg/ml) for 24 hrs. The mRNA levels of MMP‐1 and procollagen type I were determined by RT‐PCR analysis. GAPHD mRNA was used as an internal control. The band intensities were quantified by densitometry, normalized to the level of GAPDH mRNA, and calculated as the percentage of the basal response. All data are shown as the mean ± S.D. of at least three independent experiments performed in triplicate. ##*P* < 0.01, ###*P* < 0.001, compared to the control; *P < 0.05, **P < 0.01, ***P < 0.001, compared to the group receiving only UVB radiation.

The MMP‐1 and procollagen type I mRNA level was measured in UVB induced NHDFs. As shown, there was a low basal mRNA expression of MMP‐1 in quiescent fibroblasts. The MMP‐1 mRNA level was drastically elevated in UVB stimulated NHDFs. Consistent with the ELISA result, in the presence of TV (10 and 100 μg/ml), the MMP‐1 transcript expression was sharply induced by 53.3% and 63.5%, respectively, compared with that of UVB irradiated control cells. On the contrary, the mRNA level of procollagen type I was diminished in the UVB induced NHDFs. With the treatment with TV (10 and 100 μg/ml), the expression of procollagen type I was meaningfully up‐regulated by 508.4% and 652.3%, respectively, compared with that of UVB irradiated cells (Fig. 9).

### Alleviation of TV on the phosphorylation of MAPK and AP‐1 signalling pathway in UVB irradiated NHDFs

**Figure 10:**
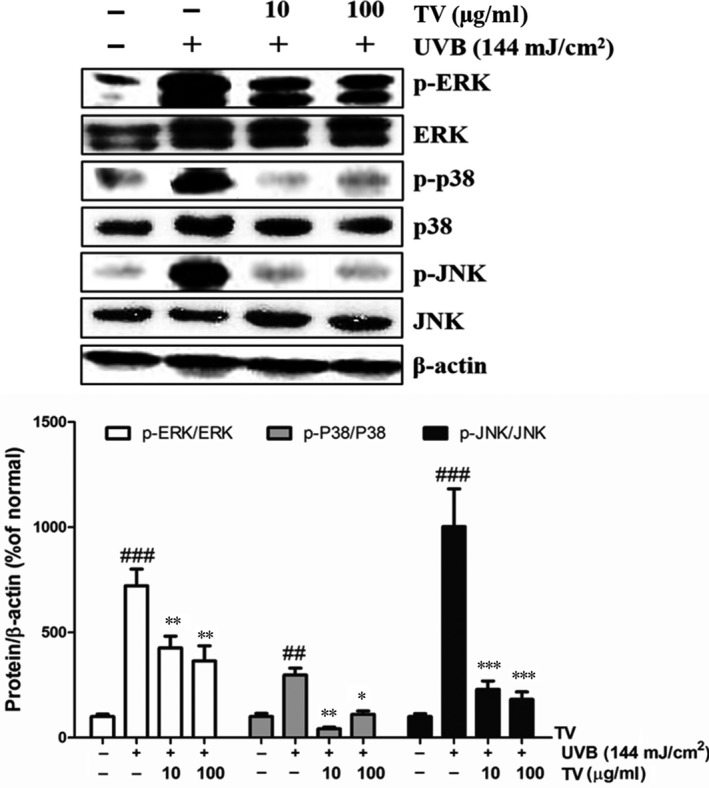
Effects of TV on MAPK activation in UVB irradiated NHDFs. NHDFs were irradiated with UVB followed by treatment with TV (10 and 100 μg/ml) for 1 hr. The phosphorylation of ERK, JNK and p38 were detected by Western blotting. β‐actin was used as an internal control. The band intensities were quantified by densitometry, normalized to the level of β‐actin, and calculated as the percentage of the basal response. The results are shown as the mean ± S.D. of at least three independent experiments performed in triplicate. ##*P* < 0.01, ###*P* < 0.001, compared to the control; *P < 0.05, **P < 0.01, ***P < 0.001, compared to the group receiving only UVB radiation.

The MAPK signalling pathways was shown to mediate the expression of MMP‐1 10. To investigate the underlying mechanism of TV worked, we assessed the phosphorylation rate of the MAPK family, consisting of ERK, JNK and p38 by Western blotting. As shown in Figure 10, UVB initiated the activation of ERK, JNK and p38, leading to drastically phosphorylation of those proteins. Whereas, TV significantly quenched UVB‐induced phosphorylation of ERK (p‐ERK), p38 (p‐p38) and kinase JNK (p‐JNK), compared to the UVB irradiated control cells. Treatment with TV (100 μg/ml) diminished 49.4% of p‐ERK, 63.1% of p‐p38 and 81.9% of p‐JNK, compared with the UVB irradiated control group, respectively (Fig. 10).

**Figure 11:**
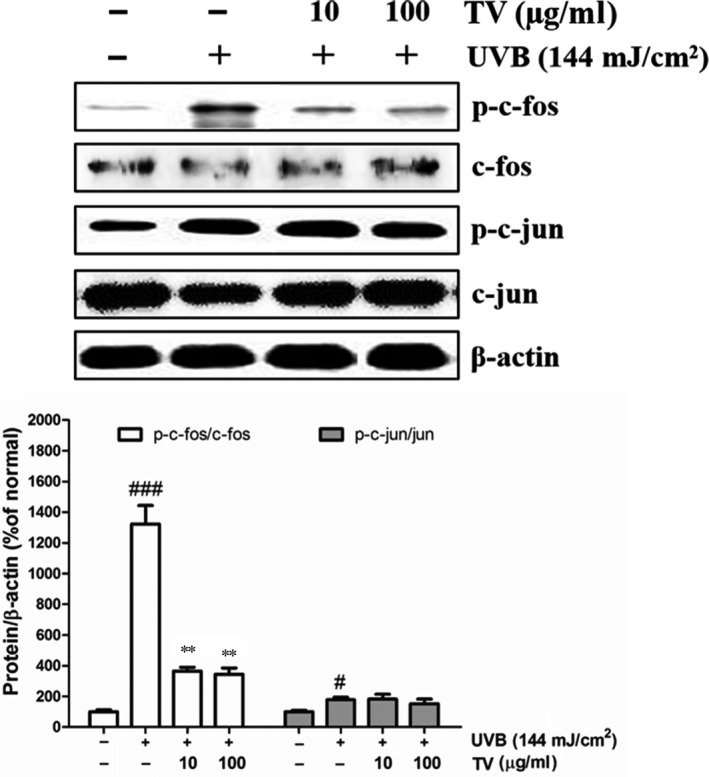
Effects of TV on AP‐1 activation in UVB irradiated NHDFs. NHDFs were irradiated with UVB followed by treatment with TV (10 and 100 μg/ml) for 4 hrs. The phosphorylation of c‐fos and c‐jun were detected by Western blotting. β‐actin was used as an internal control. The band intensities were quantified by densitometry, normalized to the level of β‐actin, and calculated as the percentage of the basal response. The results are shown as the mean ± S.D. of at least three independent experiments performed in triplicate. #*P* < 0.05, ###*P* < 0.001, compared to the control; **P < 0.01, compared to the group receiving only UVB radiation.

Activated protein‐1 is the downstream factors of MAPK. To further investigate the action of TV worked, we measured the phosphorylated forms of c‐fos (p‐c‐fos) and c‐jun (p‐c‐jun) induced by UVB in NHDFs. As shown in (Fig. 11), exposure to UVB leading to drastic elevation of p‐c‐fos and p‐c‐jun. When treated with TV, the UVB induced p‐c‐fos and p‐c‐jun production was significantly inhibited. Specifically, TV (100 μg/ml) significantly inhibited the expression of p‐c‐fos by 73.9%, and suppressed p‐c‐jun by 14.6%, compared with the UVB irradiated control group, respectively.

## Discussion

Based on epidemiological studies, increasing numbers of people are suffering from skin damage because of overexposure to solar UV irradiation. Skin chronically exposes to solar UV radiation resulting in oxidative stress, inflammation, ROS‐mediated DNA damage, disorder of cellular signalling pathways, which accelerate skin photoageing 21. Botanicals possessing antioxidant, anti‐inflammatory and immunomodulatory properties, are promising to be exploited as therapeutic agents for a variety of skin disorders, including photoageing 22. TV extract and one of its major compounds rosmarinic acid have been shown to protect human keratinocytes against UVA and UVB induced damages 23. As shown in this study, TV was effective in ameliorating UV mediated damages in NHDFs and hairless mice (Fig. 5). Further, we demonstrated that TV exerted its protective effect by activation of Nrf2‐ARE and inhibition of MAPK/AP‐1 pathway.

Extracellular matrix is directly or indirectly disturbed by UV radiation with elevated MMPs activity. The MMPs activity was increased in skin because of UV irradiation, which resulted in collagen destruction and led to photoageing 5. Histological and ultrastructural researches indicated that the enhanced epidermal thickness and alterations of connective tissue organization were ubiquitous in photodamaged skin 24. Collagen is fundamental to sustain the elasticity of skin structures 25. Matrix metalloproteinases attack and degrade collagen and elastin 26. It is believed that blocking MMPs is an effective way to prevent UV induced photodamage. In this study, our results indicated that TV inhibited UVB irradiation induced MMP‐1 production and the subsequent loss of collagen in NHDFs (Fig. 5), and also prevented proteolytic degradation of existing elastic fibres in the dermal layer of hairless mice (Fig. 5).

The level of ROS was dramatically elevated because of UVB induction, which was related to MMPs production and collagen fragmentation 27. The ROS stimulated MMPs were detected accompanying with broken connective tissue in photoaged skin 4. Considerable researches have reported that antioxidative botanicals are potential to prevent skin damages caused by UV irradiation. For example, ergothioneine executed dermato protective properties through induction antioxidant genes in UVA irradiated human keratinocytes 28. Accordingly, botanical antioxidants may be useful to ameliorate UV induced photoageing by scavenging and quenching ROS.

Ultraviolet induced ROS also accelerated the reaction of inflammatory cytokines. The cytokines, such as IL‐6, were proved to accentuate direct destructive effects on cells which accelerated photoageing 29. Thus, prevention of inflammatory reactions is an important strategy to comprehensively protect the skin against adverse effects of solar. On the other hand, TGF‐β1 is associated with type I procollagen synthesis. It has been reported that IL‐6 interfered the TGF‐β1 pathway and impaired type I procollagen synthesis by inducing MMP‐1expression, leading to collagen loss in the dermis 30, 31. In this study, our results showed that UVB irradiation provoked the secretion of IL‐6 in NHDFs, which was subsided by treatment with TV (Fig. 8). Meanwhile, TV promoted the expression of TGF‐β1 (Fig. 8). It is possible that TV elevated the level of type I procollagen by enhancing TGF‐β1 pathway.

Wrinkle occurs because of accumulated skin damages, such as matrix destruction and skin inflammation. Skin inflammation motivated MMPs, resulting in collagen degradation in the epidermis and dermis 28. The inflammatory responses in UV irradiated fibroblasts were modulated through a variety of signalling pathways, such as activation of NF‐κB, the AP‐1 complex and MAPKs 32. To investigate the underlying mechanism, we proceeded further researches on MAPK and AP‐1 signalling pathways in DHNFs. Mitogen activated protein kinase signalling pathways play crucial roles in many activations of cells, including cell proliferation, cell motility and cell death 33. Mitogen activated protein kinases mainly act as regulators of transcription factors and transmitters that relay extracellular signals to the nucleus and further trigger target gene expression. Icariin inhibited TNF‐α/IFN‐γ induced inflammatory response *via* inhibition of p38‐MAPK signalling pathway in human keratinocytes 34. Geniposide suppressed LPS‐induced nitric oxide, PGE2 and inflammatory cytokine by downregulating MAPK and AP‐1 signalling pathways in macrophages 35. In this study, our results showed that TV suppressed the phosphorylation of MAPKs and AP‐1 complex in UVB irradiated NHDFs, indicating that TV inhibited MMP‐1 production *via* MAPK and AP‐1 signalling pathways. Taken together, inflammatory factor, MAPK and AP‐1 induction are initiated by oxidative stress 36, therefore regulation of ROS levels is a primary mechanism by which botanicals could be particular effective.

Ageing and many pathological processes have been known to be associated with the alteration of energy metabolism as a result of mitochondrial damages such as oxidative stress caused by ROS 37. Mitochondrial are the cellular engines responsible for generating high energy. Dihydrolipoyl dehydrogenase, a flavoprotein enzyme, was the only protein inactivated after UVB exposure among the TCA cycle‐associated proteins. It is the integral component (E3) of the metabolic muti‐enzyme complexes, including pyruvate dehydrogenase, α‐ketoglutarate dehydrogenase and α‐keto acid dehydrogenase 38. Particularly, α‐keto acid dehydrogenase could be the primary target of ROS and regulate the deficit of mitochondrial metabolism with oxidative stress 39. Dihydrolipoyl dehydrogenase converts dihydrolipoic acid into α‐lipoic acid in the cells 40. Interestingly, α‐lipoic acid can active two major regulators of cytoprotective response to oxidative stress, Nrf2 and HO‐1 41, 42. In other words, DLD not only acts as a target of ROS, but also plays a role in cellular defense against oxidative stress. In this study, DLD was the first time investigated as a potential UVB target involved in skin ageing. The DLD protein level was decreased in NHDFs by UVB irradiation, while was recovered following treatment with TV. In addition, a specific reversible inhibitor of E3, MICA, was introduced to investigate the role of the protein in Nrf2 signalling pathway. As shown, the expression of Nrf2 was blocked as a result of exposure of MICA, which inhibited the function of DLD (Fig. 7). Namely, DLD regulated the expression of Nrf2. As known Nrf2‐ARE pathway was a critical axis to relay extracellular signals to the nucleus and further initiated antioxidant gene expression under oxidative stress 43. Our results showed that ROS significantly induced the activation of Nrf2, and TV treatment increased nuclear localization of Nrf2, resulting in the enhanced production of antioxidants, such as HO‐1 and NQO‐1. The mechanism of TV activated Nrf2 may belongs to the polyphenols disrupt the Keap1‐Nrf2 complex, probably through interaction with the thiols present on Keap1 44. However, the mechanism of TV modified the Keap1‐Nrf2 complex remain open that need further investigation to confirm.

## Conclusions

In conclusion, the present study demonstrated the photoprotective effects of TV on skin damage caused by UVB radiation. TV protected collagen breakdown *via* inhibiting MMP‐1 expression. In addition, TV mitigated accumulation of ROS induced by UVB radiation. TV augmented production of DLD, which acted as cellular defense against oxidative stress through Nrf2‐ARE signalling pathway. Besides, TV inhibited the phosphorylation of MAPKs and AP‐1 signalling pathways in NHDFs. Based on our present results, we proposed the use of TV in curtailing skin oxidative stress caused by chronic UVB exposure.

## Conflict of interest

The authors declare that there are no conflicts of interest.
